# Accelerated CO_2_ Hydration with Thermostable *Sulfurihydrogenibium azorense* Carbonic Anhydrase-Chitin Binding Domain Fusion Protein Immobilised on Chitin Support

**DOI:** 10.3390/ijms20061494

**Published:** 2019-03-25

**Authors:** Juan Hou, Xingkang Li, Michal B. Kaczmarek, Pengyu Chen, Kai Li, Peng Jin, Yuanmei Liang, Maurycy Daroch

**Affiliations:** 1 School of Environment and Energy, Peking University Shenzhen Graduate School, Shenzhen 518055, China; 1601214093@sz.pku.edu.cn (J.H.); lixk@pkusz.edu.cn (X.L.); kaczmarek.micha4@gmail.com (M.B.K.); z0988876752@gmail.com (P.C.); 1601214099@sz.pku.edu.cn (K.L.); jinpeng@pkusz.edu.cn (P.J.); yuanmeiliang@pku.edu.cn (Y.L.); 2 Institute of Technical Biochemistry, Lodz University of Technology, 90-924 Lodz, Poland

**Keywords:** carbonic anhydrase, chitin, enzyme immobilisation, chitin binding domain, CO_2_ hydration

## Abstract

Carbonic anhydrases (CAs) represent a group of enzymes that catalyse important reactions of carbon dioxide hydration and dehydration, a reaction crucial to many biological processes and environmental biotechnology. In this study we successfully constructed a thermostable fusion enzyme composed of the *Sulfurihydrogenibium azorense* carbonic anhydrase (Saz_CA), the fastest CA discovered to date, and the chitin binding domain (ChBD) of chitinase from *Bacillus circulans*. Introduction of ChBD to the Saz_CA had no major impact on the effect of ions or inhibitors on the enzymatic activity. The fusion protein exhibited no negative effects up to 60 °C, whilst the fusion partner appears to protect the enzyme from negative effects of magnesium. The prepared biocatalyst appears to be thermally activated at 60 °C and could be partially purified with heat treatment. Immobilisation attempts on different kinds of chitin-based support results have shown that the fusion enzyme preferentially binds to a cheap, untreated chitin with a large crystallinity index over more processed forms of chitin. It suggests significant potential economic benefits for large-scale deployment of immobilised CA technologies such as CO_2_ utilisation or mineralisation.

## 1. Introduction

The ever-increasing atmospheric concentration of CO_2_ and resulting climate change require development of new technologies for carbon capture, sequestration and utilisation. In recent years there has been an increased interest in the development of biomimetic approaches to carbon capture, mineralisation and conversion to more reduced forms of carbon [1,2,3].

Availability of carbon dioxide for biosequestration and bioutilisation increases when the compound is dissolved in water and converted to its hydrated form in a relatively complex and dynamic relationship between different forms of inorganic carbon species that is highly dependent on pH [4,5,6] and ionic strength [7]. The CO_2_ hydration reaction, as shown in Equation (1), is considered rate-limiting and an entry point for both carbon mineralisation and carbon reutilisation technologies with biological systems [8,9].

(1) $$\begin{array}{ccc} H^{+}+HCO_{3}^{-} & ⇌ & H_{2}CO_{3}\\ ⇃↾ & & ↿⇂\\ CO_{2} & + & H_{2}O \end{array}$$

Carbonic anhydrases are zinc metalloenzymes that catalyse carbon dioxide hydration and dehydration. These biocatalysts are known to be one of the fastest biocatalysts in nature, with turnover numbers in millions of reactions per second [9,10,11,12] and as such could potentially make a massive contribution to the reduction of CO_2_ emissions if employed properly. Research on these remarkable proteins goes beyond environmental applications. Core research to date belongs to the fields of drug design and pharmacology, where these enzymes are important drug targets for an array of diseases such as oedema, glaucoma, obesity, cancer, epilepsy and osteoporosis [13,14]. Developing specific inhibitors of these enzymes can have profound effects for finding cures for these diseases [13,14]. In recent years research has been also conducted for activators of these enzymes that could have a significant impact on both their environmental and medical applications [13,15] and could shed more light on important mechanisms, such as invasion, colonisation and pathogenicity of certain bacteria, all of which involve carbonic anhydrase action [15].

Among carbonic anhydrases (CA), an α-carbonic anhydrase of thermophilic bacterium *Sulfurihydrogenibium azorense* (Saz_CA) originally isolated from terrestrial hot springs of the Azores is known to be the fastest carbonic anhydrase with turnover number k_cat_ of 4.4 × 10^6^ s^−1^ [12]. In addition to direct application of CA in hydration of CO_2_, these enzymes can have a number of other applications in environmentally-friendly technologies, for example in enzymatic reactive absorption [16,17,18], CO_2_ mineralisation [1], biomimetic membranes [19] and many others. The widespread deployment of these enzymes is hindered, however, with their significant cost and problematic recycling [20].

Enzyme immobilisation has been long known as an effective method of biocatalyst reuse and recycling. Among carriers for these biocatalysts, chitin and chitosan are often used due to their high affinity towards proteins in general [21]. Since unspecific bonding of proteins to chitin and chitosan support is primarily based on weak interactions such as electrostatic, hydrogen bonding and Van der Waals forces, the binding is not very selective and results in binding numerous proteins to the support. In order to increase the specificity of binding and enzyme load per unit of carrier, tighter and more specific binding is favourable. To explore this possibility, application of efficient and specific chitin binding domains can be employed. Such domains can enhance both specificity and immobilisation strength as these types of bonding are very specific and durable [22,23].

Here we describe the expression of codon-optimised carbonic anhydrase of *Sulfurihydrogenibium azorense* fused with a chitin binding domain (ChBD) of *Bacillus circulans* chitinase A1 (chiA) in *Escherichia coli*, a simplified protein purification scheme based on thermal precipitation, assessment of ChBD binding properties to different chitin-based supports and the fusion domain effect on the enzyme properties.

## 2. Results and Discussion

### 2.1. Expression and Purification of Saz_CA Variants

**Figure 1:**
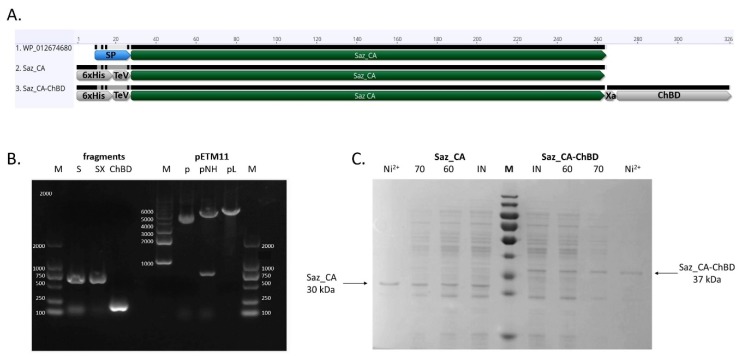
Construction and expression of *Sulfurihydrogenibium azorense* carbonic anhydrase (Saz_CA) variants. (**A**) Overview of the constructs made in this study: WP_012674680—native sequence of Saz_CA containing original signal peptide; Saz_CA—mature sequence of Saz carbonic anhydrase with N-terminally fused 6× His tag and TeV protease digestion site; Saz_CA-ChBD—mature sequence of Saz_CA with N-terminally fused 6× His tag TeV protease digestion site, and C-terminally fused chitin binding domain (ChBD) and factor Xa protease site. (**B**) Agarose gel electrophoresis of DNA fragments used to construct expression plasmids pETM11_Saz_CA and pETM11_Saz_CA-ChBD: M—molecular weight markers, S—Saz_CA fragment amplified with Saz_CA_pETM11_F and Saz_CA_pETM11_R, SX—Saz_Xa fragment amplified with Saz_CA_pETM11_F and Saz_CA_XaFusion_R, ChBD—chitin binding domain amplified with Saz_CA_XaFusion_ChBD_F and ChBD_pETM11_R, p—pETM11 expression plasmid, pNH—pETM11 expression plasmid digested with NcoI and HindIII, pL—linearised purified NcoI and HindIII digested pETM11 expression plasmid. (**C**) SDS-PAGE showing expression and purification of Saz carbonic anhydrase variants. Saz_CA: IN—*Escherichia coli* BL21(DE3) harbouring Saz_CA construct induced with IPTG, 60—proteins remaining in the crude lysate after 30 min incubation at of Saz_CA lysate at 60 °C, 70—proteins remaining in the crude lysate after 30 min incubation of Saz_CA lysate at 70 °C, Ni^2+^—proteins eluted with imidazole from Ni^2+^ affinity chromatography column. Saz_CA-ChBD: IN—*E. coli* BL21(DE3) harbouring Saz_CA-ChBD construct induced with IPTG, 60—proteins remaining in the crude lysate after 30 min incubation at of Saz_CA-ChBD lysate at 60 °C, 70—proteins remaining in the crude lysate after 30 min incubation of Saz_CA-ChBD lysate at 70 °C, Ni^2+^—proteins eluted with imidazole from Ni^2+^ affinity chromatography column. M—molecular weight marker (size range: 180, 130, 100, 70, 55, 40, 35, 25, 15, 10 kDa).

The two variants of thermostable *Sulfurihydrogenibium azorense* carbonic anhydrase (Saz_CA), both N-terminally fused to a hexahistidine tag and TeV protease site—one additionally containing an Xa protease cleavage site and chitin binding domain extracted from *Bacillus circulans* WI-12 chitinase A1 (chiA) gene—have been successfully expressed as soluble proteins in *Escherichia coli* BL21(DE3), as shown in Figure 1A. Analysis of the gene expression products using SDS-PAGE revealed major bands of Saz_CA, as shown in Figure 1B (30 kDa), and Saz_CA-ChBD, as shown in Figure 1B (37 kDa), in soluble fraction of *E. coli* lysates.

Purification using a nickel affinity column resulted in near homogenous protein preparation, as show in Figure 1B. Simultaneous attempts to purify both proteins with a single step heat treatment at 60 °C and 70 °C were performed, resulting in a satisfactory degree of purification of both constructs, as shown in Table 1 and Table 2. Whilst the nickel affinity column resulted in higher sample purity and the proteins were used for subsequent studies of enzyme variant characterisation, the simplicity of a single step heat treatment, combined with high purity of resultant protein, is valuable for the future when large quantities of enzyme will be required for a scaled-up study. Additionally, it was found that after 30 min of thermal purification of Saz_CA-ChBD construct at both 60 °C and 70 °C, the ChBD tag retained the ability to bind to the chitin support, providing another alternative route for purification with affinity chromatography (see Section 2.3 below). These results correspond to prior studies on independent fragments of this construct. It was previously mentioned that Saz_CA can be partially purified with heat treatment [12] and that *B. circulans* ChBD exhibits relatively high thermostability and can be used as an affinity tag for thermostable enzymes [24]. Our results confirm these findings and prove applicability of such a fusion construct for a streamlined purification protocol based on heat precipitation and affinity adsorption on chitin support.

### 2.2. Properties of Saz_CA Variants

**Figure 2:**
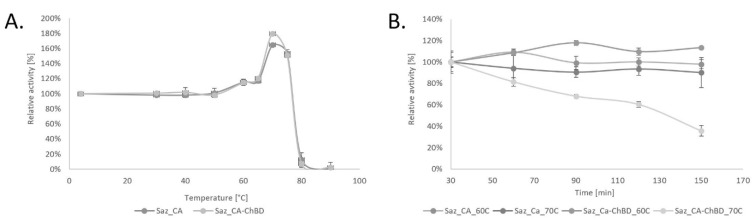
Thermostability of Saz_CA variants. (**A**) Thermostability of Saz_CA variants during 30 min incubation at different temperatures. (**B**) Thermostability time-course during incubation of Saz_CA variants at 60 °C and 70 °C for up to 150 min.

Nickel column purified variants of Saz_CA and Saz_CA-ChBD were assessed for basic parameters such as thermostability and effects of ions and inhibitors to verify the effect of ChBD on the properties of Saz_CA. These results are summarised in Figure 2 and Table 1 and Table 2. During the course of these studies it was found that both constructs undergo thermal activation at temperatures exceeding 60 °C. Enzyme thermal activation has not been previously discussed, and our data do not support prior reports that show linear trends of thermostability using a similar assay [12]. Interestingly, these authors’ similar work on a related carbonic anhydrase of *Sulfurihydrogenibium yellowstonense* (Ssp_CA) [25,26] seems to follow the trend we observed in our study, i.e., 100% thermostability until the critical temperature of 50 °C was achieved. This was followed by a rapid rise of activity in excess of 100%. In the case of this study, these values are 165% and 180% at 70 °C for Saz_CA and Saz_CA-ChBD, respectively. These data have not been extensively discussed to date. We believe that observed results are due to heat-induced changes in the three-dimensional conformation of the protein. Both enzymes Saz_CA and Ssp_CA originate from thermophilic microorganisms that inhabit terrestrial hot springs and typically grow at temperatures exceeding 70 °C [27]. CO_2_ hydration assay is typically performed at 0 °C to maximise the CO_2_ solubility in water and saturation of the enzyme with substrate [28]. Also, the original enzyme used for the assay developed by Roughton and Booth (1945) was human blood carbonic anhydrase, a typical mesozyme of optimal temperatures in the mesophilic range and therefore unlikely to undergo such activation at higher temperatures. We believe that both enzymes (Saz_CA and Ssp_CA) undergo structural shifts at 50 °C, probably surface exposing the active site and resulting in observed increased activity. Due to the nature of the assay, i.e., rapid quenching of incubated proteins on ice before assessment of residual enzymatic activity with CO_2_ hydration assay, the more active conformation of the enzyme obtained during heating above 50 °C is retained, which results in increased activity. Although we could not identify similar reports about carbonic anhydrases in the literature, the phenomenon of similar activation is known in enzymology. For example, lipases undergo a structural shift in the presence of hydrophobic substrates, [29] whilst piezozymes undergo activation at high pressures [30]. Most recently a thermophilic esterase of *Pyrococcus furiosus* was found to be thermally activated through such changes of conformation resulting in heat-induced formation of dimers [31]. The exact structural causes of thermal activation of Saz_CA are yet to be elucidated and our preliminary data suggest that dimerisation is not a mechanism that is found in Saz_CA activation. The presumed thermal activation of the enzyme is interesting from a biological perspective and is a promising finding considering application of Saz_CA for CO_2_ hydration from industrial sources that typically have high temperatures [32]. Interestingly, to date Saz_CA has not been applied for CO_2_ hydration reaction above 0 °C. This most likely is due to problems associated with interpretation of the assay at elevated temperatures, as solubility of CO_2_ in water drastically drops and the enzyme may no longer be saturated with substrate, especially considering increased rates of reaction at high temperatures.

Another important finding from our study is that at peak temperatures, i.e., 70 °C, both constructs showed comparable parameters, as shown in Figure 2A, indicating that adding ChBD did not negatively impact the activity-temperature profile of the Saz_CA, which combined with favourable binding properties to various forms of chitin makes this fusion protein applicable for immobilisation on different chitin-based supports. On the other hand, the Saz_CA-ChBD construct incubated at 70 °C for over 30 min demonstrated considerable loss of enzymatic activity, which the Saz_CA did not exhibit. It is likely that under prolonged incubation at 70 °C the denaturation of the fusion partner takes place and the enzyme loses its activity, as shown in Figure 2B. This limits the application of the Saz_CA-ChBD protein to 60 °C until a new, more thermostable variant of the ChBD is available.

Inhibitor-effects analysis (Table 3) shows that ChBD has no effect on the fusion protein enzymatic properties, with data concerning the inhibitory effect of sulphanilamide and acetazolamide being identical for both constructs. Further, the effects of important anions such as HCO_3_^−^, CO_3_^2−^ and SO_4_^2−^ are identical and negligible for both constructs tested and in accordance with previous studies on Saz_CA [12,33]. However, the effects of cations on enzymatic activity of Saz_CA have not been reported to date.

Our results show that both constructs demonstrate a pattern of interactions similar to previously reported effects for other thermostable bacterial carbonic anhydrases (i.e., inhibition by zinc, copper, iron and manganese) and lack of effect of the first group of metals such as sodium and potassium and no effect of calcium [3,34,35,36]. More diverse results were obtained with magnesium, which is typically considered as having either no effect on enzymatic activity [35,36] or being a slight inhibitor [3,34]. Our results show inhibited enzymatic activity for Saz_CA. This effect was eliminated in the fusion protein containing the chitin binding domain. Since there are no reports on the effect of magnesium on ChBD of *Bacillus circulans* WI-12 chitinase A1, and results of the effect of this metal on other chitinases conflict, a follow-up study is required to elucidate the effect of the metal on the domain and its application as a fusion partner.

### 2.3. Immobilisation of Saz_CA Variants on Chitin Support

The efficiency of different chitin supports for the immobilisation of Saz_CA-ChBD were tested. Interestingly, it was shown that untreated chitin had the highest immobilisation efficiency among all supports, 82.9%. Although commercial chitin resin had similar immobilisation efficiency (80.9%) to the untreated chitin, the activity of the immobilised enzyme was 60% lower compared with the untreated chitin, as shown in Table 4. Both the immobilisation efficiency and activity of the immobilised enzyme for pre-treated chitin were significantly lower than those of the other two materials. Adding the chitin binding domain (ChBD) was essential to efficient binding to the support, as the Saz_CA construct was unable to bind to the commercial chitin resin, indicating that fusion protein immobilisation was predominantly due to including the chitin binding domain and its interaction with support.

**Figure 3:**
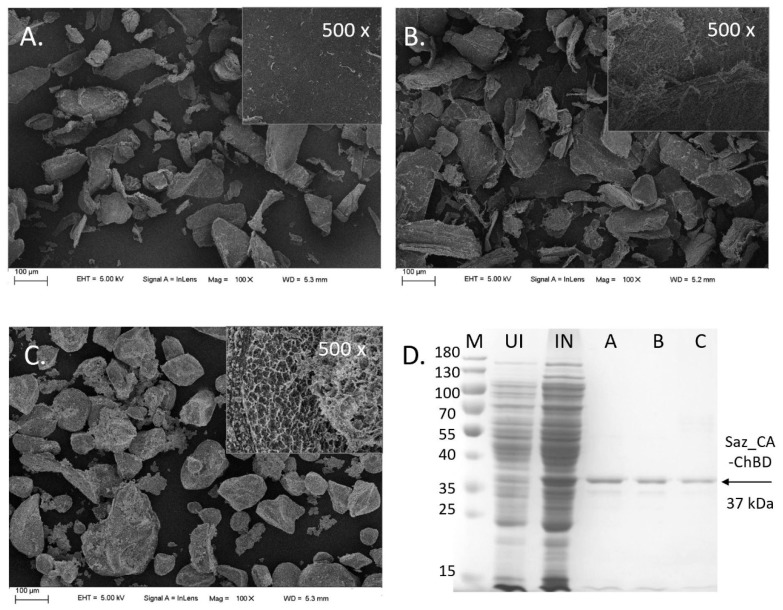
Saz_CA-ChBD immobilised on different chitin supports. (**A**–**C**) Scanning electron microscope (SEM) images of different chitin supports used for immobilisation of Saz_CA-ChBD. (**A**) Untreated chitin. (**B**) Pre-treated chitin. (**C**) Commercial chitin resin. (**D**) SDS-PAGE profile of proteins eluted from each support (**A**–**C**), M—molecular weight marker (size range: 180, 130, 100, 70, 55, 40, 35, 25, 15, 10 kDa), UI—*E. coli* BL21(DE3) harbouring Saz_CA-ChBD construct, IN—*E. coli* BL21(DE3) harbouring Saz_CA-ChBD construct induced with IPTG, A—elution profile from untreated chitin, B—elution profile from pre-treated chitin, C—elution profile from commercial chitin resin.

Analysed SEM images indicate that all chitin supports had similar particle sizes of 10–100 μm. However, the commercial chitin resin was much more porous than the other two materials, as shown in Figure 3, which in principle should have facilitated the immobilisation of the enzyme. Crystallinity indices (CrI) analysis of different supports suggests an alternative explanation: the CrI of untreated chitin, commercial chitin beads and pre-treated chitin, were 98.8%, 90.1% and 81.6%, respectively, as shown in Table 5. It was previously reported that the chitin binding domain of *B. circulans* chitinase A used in this study preferentially binds to highly crystalline regions of chitin through tryptophan residue located in the cleft formed between two β-sheets [23,37]. It is therefore reasonable that a higher degree of crystallinity of the untreated chitin facilitated the immobilisation of Saz_CA-ChBD, and less crystalline forms such as pre-treated chitin and commercial resin preparation were not as effective. Additionally, the commercial chitin preparation’s highly porous structure could have a negative effect on mass transfer between the gas phase and liquid phase and, subsequently, on the availability of substrate at the active site of the enzyme resulting in lower observed enzymatic activity. Commercial chitin resin used for enzyme immobilisation is usually prepared by dissolving crystalline chitin in LiCl-DMAc or NaOH-urea [38]. The as-obtained gels are then contacted with anti-solvent to regenerate chitin. These processes utilise large quantities of organic solvents and result in poor yields of final chitin resin, making applications of less processed chitin desirable.

**Figure 4:**
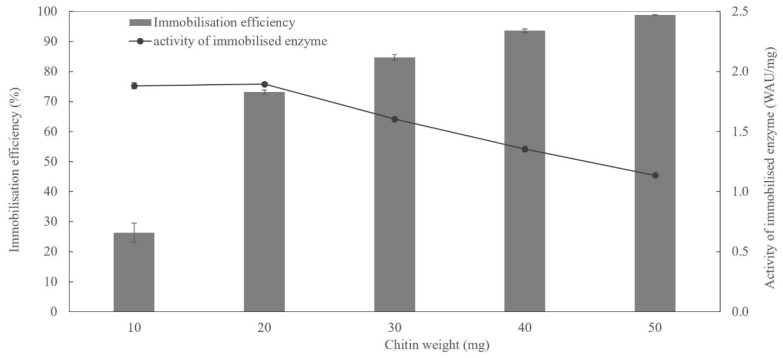
Effect of chitin weight on immobilisation efficiency and enzymatic activity of Saz_CA-ChBD immobilised on untreated chitin.

The ratio of chitin-to-enzyme significantly affected the immobilised enzyme’s immobilisation efficiency and its activity. Efficiency increased proportionally from 26.4% to 98.9% with raising chitin weight from 10 to 50 mg. Meanwhile, as more chitin was used for immobilisation and enzyme saturation of the support increased, the immobilised enzyme’s specific activity gradually decreased, most likely due to issues with substrate diffusion to the enzyme, as shown in Figure 4. Our results show that when using ChBD fusion constructs, a much simpler and cheaper alternative, i.e., untreated chitin, is better suited than commercial resin, especially for environmental applications where large-scale enzyme deployment is required, and costs of enzyme preparation are of paramount importance.

## 3. Materials and Methods

### 3.1. DNA Manipulations and Construction of Vectors

The full-length sequence of *Sulfurihydrogenibium azorense* Az-Fu1 gene encoding thermophilic carbonic anhydrase Saz_CA [12] was extracted from the genome of the strain deposited in the NCBI with accession number CP001229. The translated gene product was analysed for the presence of signal peptide with Signal IP 4.0, and the mature amino acid sequence was reverse translated into a nucleotide sequence optimised for the expression in *Escherichia coli*. The nucleotide sequence of the chitin binding domain (156 bp) (ChBD) was extracted from *Bacillus circulans* WI-12 chitinase A1 (chiA) [39] deposited in the NCBI with accession number M57601.1. Both sequences were synthesised by a commercial supplier (Genewiz, Suzhou, China) and delivered cloned into pUC57 vectors. These fragments were subsequently used for construction of expression vectors based on pETM11 (EMBL Heidelberg). The construct expressing free, N-terminal His tagged Saz_CA was performed as follows: the backbone vector pETM11 was linearised with NcoI and Hind III restriction enzymes (Thermo Scientific, Waltham, MA, USA); Saz_CA gene was amplified with Saz_CA_pETM11_F TTTATTTTCAGGGCGCCATGGAACACGCAATTCTGCAG and Saz_CA_pETM11_R GTGCGGCCGCAAGCTTTAGTTAGATTCCAGGATGTAA primer pair and Phusion pfu DNA polymerase (NEB, Ipswich, MA, USA). A construct expressing C-terminally bound ChBD-fused, N-terminal His tagged Saz_CA was performed as follows: the backbone vector pETM11 was linearised with NcoI and Hind III restriction enzymes (Thermo Scientific); Saz_CA gene was amplified with Saz_CA_pETM11_F TTTATTTTCAGGGCGCCATGGAACACGCAATTCTGCAG and Saz_CA_XaFusion_R GTTAGATTCCAGGATGTAA primer pair; the Chitin-binding domain (ChBD) was amplified with Saz_CA_XaFusion_ChBD_F ATCCTGGAATCTAACATCGACGGTCGTGCACTTACGACAAATCCTGGTGTATCC and ChBD_pETM11_R CTCGAGTGCGGCCGCATTAGCCCAGTGCACCTTGAAGCTGCCACAAGGC primer pair using Phusion DNA polymerase (NEB, USA). All PCR amplifications were performed according to manufacturer’s instructions using HF Buffer and the following temperature regime: 98 °C (30 s), then 30 cycles of 98 °C (10 s), 64 °C (30 s), 72 °C (35 s) and final extension 72 °C (10 min). The resultant PCR products were purified using a DNA Clean and Concentrator Kit (Zymo Research, Irvine, CA, USA), their concentrations were adjusted to be equimolar, and final constructs were assembled using a ClonExpress Ultra One Step Cloning Kit (Vazyme, Nanjing, China) and directly transformed to *E. coli* DH5α (Tiangen, Beijing, China) according to manufacturer’s instructions. Successful assembly of two constructs (pETM11_SazCA and pETM11_SazCA_ChBD) was confirmed with colony PCR and sequencing by Guangzhou IGE Biotechnology (Guangzhou, China).

### 3.2. Protein Expression and Purification

Two recombinant plasmids pETM11_Saz_CA and pETM11_Saz_CA-ChBD were transformed into *E. coli* BL21(DE3) pLysS (Tiangen, Beijing, China), according to manufacturer’s instructions. The transformed cells were cultured in LB medium containing 30 mg L^−1^ kanamycin and 30 mg L^−1^ chloramphenicol until OD_600_ reached 0.7, induced with IPTG added to a final concentration of 0.5 mM; induction was carried out for 9 h. Cells were pelleted by centrifugation, resuspended in 20 mM Tris-HCl pH 8.0 and lysed by sonication on ice for 10 min (SCIENTZ-ⅡD, Ningbo, China). The power regime was set as follows: 50% power, pulse 5 s, break 9 s. Resultant lysate was centrifuged to collect the supernatant, crude enzyme solution. Crude enzyme solution was loaded onto a nickel affinity column (CWBIO, Beijing, China) pre-equilibrated with binding buffer (20 mM Tris-HCl pH 7.9, 10 mM imidazole and 0.5 M NaCl) until no protein was detected in the flow-through. The protein bound to the column was eluted with elution buffer (20 mM Tris-HCl pH 7.9, 500 mM imidazole and 0.5 M NaCl). Alternatively, two proteins were purified using heat treatment, i.e., cell lysates incubated at 60 or 70 °C for 30 min and subsequently quenched on ice and centrifuged at 10,000 g for 10 min to pellet denatured proteins. The purity of the Saz_CA and Saz_CA-ChBD was analysed by SDS-PAGE [40], using 12% resolving gel. Protein content was quantified spectrophotometrically at 562 nm using a BCA Protein Assay Kit (Pierce) using bovine serum albumin as standard.

### 3.3. Enzyme Immobilisation

#### 3.3.1. Preparation of Chitin Immobilisation Supports

Three different chitin materials were tested for enzyme variant immobilisation. Commercial chitin powder (Aladdin, Shanghai China) was successively sifted in 100 mesh and 400 mesh sieves. The fraction with 100–400 mesh particle size was collected and denoted as untreated chitin. A second variant of treatment was used to remove residual minerals and pigments from untreated chitin. First, untreated chitin was treated with 1 M NaOH solution at 80 °C water bath for 3 h. The suspension was filtered and washed with distilled water to remove residual alkali. The resultant chitin powder was treated with 1 M hydrochloric acid solution for 12 h and washed with distilled water to neutralise pH. These steps were repeated twice. The chitin powder was lyophilised for 48 h and denoted as pre-treated chitin. The third variant used commercial chitin resin purchased from New England Biolabs (S66561L). To collect the resin stored in 20% ethanol, a 5 mL suspension was centrifuged at 8000 rpm for 2 min. The pellet was washed with 5 mL deionised water twice, lyophilised for 48 h and denoted as commercial resin.

#### 3.3.2. Enzyme Immobilisation on Chitin Supports

Twenty-three milligrams of untreated chitin, pre-treated chitin and commercial chitin resin were added into 1 mL Saz_CA-ChBD enzyme solution with known protein concentration and activity. The mixture was incubated at 4 °C, 100 rpm for 1 h. After that, it was centrifuged at 4000 rpm for 2 min. The supernatant was collected, and protein concentration and enzyme activity were determined. The pellet fraction was washed with binding buffer (500 mM NaCl, 20 mM Tris-HCl, 1 mM EDTA, 0.1% Tween-20, pH 8.0) until no CA activity was detected in the solution. After being lyophilised for 48 h, the CA activity of immobilised enzyme was determined. At the same time, 20 mg of the three types of immobilised enzyme was analysed with SDS-PAGE. To test the effect of ChBD fusion on the efficiency of immobilisation, commercial chitin resin was added into 1 mL Saz_CA solution with other conditions unchanged. To investigate the effect of support weight on Saz_CA-ChBD immobilisation efficiency, 10 to 60 mg untreated chitin powder was used.

Immobilisation efficiency is defined as follows:

Immobilisation efficiency (%) = (1 − A/B) × 100, where A is the activity of the enzyme solution after immobilisation and B is the activity of the supernatant before immobilisation [41].

#### 3.3.3. Analysis of Chitin Immobilisation Supports

Untreated chitin, commercial chitin bead and pre-treated chitin were characterised by X-ray diffractograms (XRD) and scanning electron microscopy (SEM), whilst presence of Saz_CA-ChBD was confirmed using activity assay (see Section 3.4 below) and SDS-PAGE. XRD were carried out on a Bruker AXS D8 Advance X-ran diffractometer (Bruker, Karlsruhe Germany) operated at 45 kV and 100 mA with Cu Kα1 radiation at k 1.54184 A°, acceptance slot at 0.1 mm and scattering range (2 h) of 5–50° in steps of 0.1°. The crystalline index (CrI_020_; %) was calculated by: CrI_020_ = (I_020_ − I_am_) 100/I_020_, where I_020_ is the maximum intensity below 13° and I_am_, the intensity of amorphous diffraction at 16°. Degree of deacetylation (DD) was calculated by the equation based on CrI_020_ proposed before [42]. SEM was performed with a ZEISS SUPRA^®^ 55 scanning electron microscope (Carl Zeiss, Jena, Germany) operated at 10 keV with magnifications of 100×.

Immobilised protein was eluted from each of the supports in the following manner: 20 mg of each lyophilised support containing Saz_CA-ChBD was incubated in 30 μL of SDS-PAGE loading buffer containing β-mercaptoethanol and boiled for 10 min. The mixture was centrifuged and 10 μL of the supernatant was loaded on the gel, electrophoresis was performed as described in Section 3.2 above.

### 3.4. CO_2_ Hydration Assay

Enzymatic activity of Saz_CA variants was performed using CO_2_ hydration assay and presented as Wilbur–Anderson units (WAU) [43] using methods originally devised by Roughton and Booth [28]. Since CO_2_ hydration assay results in formation of bicarbonate ions and protons, it results in a pH shift that can be monitored using bromothymol blue as an indicator. The indicator is yellow at pH lower than 6.0 and blue when pH is higher than 7.6. The protocol for the estimation of enzymatic activity was adapted from Capasso et al. [44] and executed as follows: 1 mL of ice-cold 25 mM Tris-SO_4_ pH 8.3 containing 0.2 g L^−1^ bromothymol blue was kept on ice, and 10 μL of enzymatic solution and 1 mL of ice-cold CO_2_-saturated water were subsequently added to the buffer, and the stopwatch was started. The CO_2_ hydration activity was calculated as one Wilbur–Anderson unit (WAU), i.e., time required in seconds for a saturated CO_2_ solution to lower the pH of 0.012 M Tris-SO4 buffer from 8.3 to 6.3 at 0 °C, one WAU = (To − T)/T where To and T are the time needed for colour change from blue (pH 8.3) to yellow (pH 6.0) in the absence and presence of catalyst, respectively. The CO_2_-saturated solution was prepared by bubbling CO_2_ into ultrapure water on an ice bath for at least 1 h. All activity measurements were performed in triplicates.

### 3.5. Protein Properties

#### 3.5.1. Thermostability of Saz_CA Variants

Homogenous protein preparations of Saz_CA variants were incubated at the following temperatures for 30 min: 30 °C, 40 °C, 50 °C, 60 °C, 70 °C, 75 °C, 80 °C, 90 °C and subsequently quenched on ice. Residual enzymatic activity was subsequently analysed with a CO_2_ hydration assay as described in Section 3.4 above and compared against the control sample maintained at 4 °C.

#### 3.5.2. Time Course Thermostability of Saz_CA Variants

Metal affinity chromatography-purified protein preparations of Saz_CA and Saz_CA-ChBD were incubated at temperatures: 60 °C and 70 °C for 20, 60, 90, 120, 150 and 180 min. and subsequently quenched on ice. Residual enzymatic activity was analysed with a CO_2_ hydration assay as described in Section 3.4 above and compared against the control sample maintained at 4 °C.

#### 3.5.3. The Effect of Salts and Inhibitors on Saz_CA Variants

The following salts and inhibitors were added to the enzyme preparations to a final concentration of 1 mM: FeCl_3_, ZnCl_2_, MnCl_2_, CaCl_2_, KCl, NaCl, MgSO_4_, CuSO_4_, NaHCO_3_, Na_2_CO_3_, NaSO_4_, sulphanilamide and acetazolamide. The mixtures were incubated for 30 min at room temperature and tested for activity against untreated samples with CO_2_ hydration assay as described in Section 3.4 above.

## 4. Conclusions

We constructed a thermostable fusion enzyme composed of the *S. azorense* CA and *B. circulans* chitinase ChBD. Introduction of ChBD to the Saz_CA had no impact on the effect of ions or inhibitors on CO_2_ hydration and a protective effect from inhibition with magnesium. The fusion protein exhibited no negative effects during operation at 60 °C, whilst prolonged incubation at 70 °C resulted in some loss of activity, indicating 60 °C as the current operational limit of this construct. An immobilisation study showed that fusion protein preferentially binds to unprocessed, highly crystalline chitin over more elaborated forms of support, suggesting significant potential economic benefits of this construct for large-scale deployment.

## Figures and Tables

**Table 1:** Protein purification results of Saz_CA. WAU: Wilbur–Anderson unit.

No.	Purification Step	Enzyme Activity [WAU mL^−1^]	Total Activity [WAU]	Protein Concentration [mg L^−1^]	Specific Activity [WAU mg_prot_^−1^]	Purification Fold	Yield
**1**	Saz_CA Crude lysate	312.33	46,849	598.68	521.69	1.00	100%
**2**	Saz_CA Heat treatment 60 °C 30 min	478.28	71,741	544.39	878.55	1.68	153%
**3**	Saz_CA Heat treatment 70 °C 30 min	505.91	75,887	311.46	1624.36	3.11	162%
**4**	Saz_CA Ni^2+^ affinity chromatography	300.79	30,079	35.19	8546.74	16.38	64%

**Table 2:** Protein purification results of Saz_CA-ChBD.

No.	Purification step	Enzyme Activity [WAU mL^−1^]	Total Activity [WAU]	Protein Concentration [mg L^−1^]	Specific Activity [WAU mg_prot_^−1^]	Purification Fold	Yield
**1**	Saz_CA-ChBD Crude lysate	132.73	19,909	586.11	226.45	1.00	100%
**2**	Saz_CA-ChBD Heat treatment 60 °C 30 min	263.61	39,542	553.75	476.04	2.10	199%
**3**	Saz_CA-ChBD Heat treatment 70 °C 30 min	196.75	29,513	345.42	569.60	2.52	148%
**4**	Saz_CA-ChBD Ni^2+^ affinity chromatography	152.26	15,227	45.36	3357.09	14.82	76%

**Table 3:** Effect of ions and inhibitors (all 1 mM final) on enzymatic activity of Saz_CA variants.

Variant	Saz_CA	Saz_CA-ChBD
**Control**	100 ± 10%	100 ± 3%
**Sulphonamide**	36 ± 4%	37 ± 1%
**Acetazolamide**	0 ± 2%	0 ± 4%
**HCO_3_^−^**	90 ± 1%	96 ± 6%
**CO_3_^2−^**	99 ± 12%	96 ± 5%
**SO_4_^2−^**	97 ± 4%	93 ± 1%
**Zn^2+^**	49 ± 6%	45 ± 3%
**Mn^2+^**	82 ± 4%	81 ± 4%
**Cu^2+^**	20 ± 4%	24 ± 3%
**Mg^2+^**	81 ± 6%	101 ± 4%
**Ca^2+^**	98 ± 3%	97 ± 4%
**K^+^**	93 ± 5%	100 ± 2%
**Na^+^**	105 ± 14%	105 ± 5%

**Table 4:** Effect of different kinds of chitin on immobilisation efficiency and catalytic efficiency of Saz_CA-ChBD.

Support	Untreated Chitin	Pre-Treated Chitin	Commercial Chitin Resin
**Immobilisation Efficiency [%]**	82.88	66.64	80.88
**Activity of Immobilised Enzyme [WAU mg_chitin_^−1^]**	0.96	0.69	0.60

**Table 5:** Crystallinity index (CrI) and degree of deacetylation of different chitin supports.

Support	CrI_020_ (%)	Degree of Deacetylation
**Untreated Chitin**	98.8	6.916
**Pre-Treated Chitin**	81.6	29.740
**Commercial Chitin Bead**	90.1	18.417

## Abbreviations

CA	Carbonic Anhydrase
ChBD	Chitin Binding Domain
NCBI	National Center for Biotechnology Information
WAU	Wilbur–Anderson Unit
XRD	X-Ray Diffraction
